# Lacertus Fibrosus Syndrome: A Case Report

**DOI:** 10.7759/cureus.47158

**Published:** 2023-10-16

**Authors:** Pranav Gupta, Dhananjay Gupta, Sandeep Shrivastav

**Affiliations:** 1 Orthopaedics, Datta Meghe Institute of Higher Education and Research, Wardha, IND; 2 Orthopaedics, Fortis Flt. Lt. Rajan Dhall Hospital, New Delhi, IND

**Keywords:** carpal tunnel, cubital fossa, entrapment neuropathy, lacertus fibrosus, median nerve

## Abstract

Lacertus fibrosus syndrome is described as compression of the median nerve, which takes place beneath a layer of ligamentous tissue (lacertus fibrosus, also known as bicipital aponeurosis) slightly beyond the elbow joint. Both sexes can develop lacertus fibrosus syndrome, most often after the age of 35. The possible risk factors are repetition of movements, overwork, and manual work while the forearm is pronated. Lacertus fibrosus syndrome presents a distinct diagnostic challenge because it is a somewhat unknown and non-documented disease. Its symptoms are often mistaken for those of carpal tunnel syndrome, which complicates the differential diagnosis and management of the patient. All patients who report tingling, numbness, loss of strength, muscle loss, manual endurance, or dexterity should be investigated and tested for both carpal tunnel syndrome and lacertus syndrome. Here, a case of a 43-year-old woman is discussed, who presented with chief complaints of pain and tingling sensation in the left upper limb, which was associated with loss of thumb pinch grip. The pain was aggravated with elbow extension and relieved with rest. The patient underwent left elbow median nerve decompression and was discharged in steady condition. This case report highlights the accurate clinical presentation and surgical intervention for the syndrome, for which the outcome turned out to be satisfying.

## Introduction

In 1951, Seyfarth first defined lacertus fibrosus syndrome as pronator syndrome, a nerve compression involving the ulnar and humeral heads of the pronator teres (PT) that typically presents as a fibrous band [1]. A possible explanation for the lacertus fibrosus' dynamic compression over the median nerve is its dynamic biomechanical function in force transmission at the time of lever arm adjustment, supination restraint, and elbow flexion [2,3].

Bennett originally identified its signs in baseball pitchers in 1959 [4]. Hagert and Lalonde [5] have recently brought it to light. Loss of fine motor skills, loss of key and pinch strength, and clumsiness are typical signs of the syndrome. According to Hagert, people with lacertus fibrosus syndrome report pain when pressed on the nerve at the level of the lacertus fibrosus, weakness in muscles that are innervated by the median nerve distal to the lacertus fibrosus, and a positive scratch collapse test. Paresthesia in the hand with median nerve innervation is uncommon in these patients [2].

Ultrasonography (USG) assessment of the median nerve can be employed to determine probable compression neuropathy as well as to facilitate perineural injections. If sufficiently severe, transitions in muscle perfusion and nerve caliber can aid in the diagnosis of median nerve neuropathies [6-9]. In atypical scenarios, magnetic resonance imaging (MRI) may be valuable in differentiating between compression and neuralgic amyotrophy and also in detecting rare tumors [10-13]. Fascicular (or hourglass) constrictions of atypical cause, possibly due to trauma, inflammation, or an inflammatory condition, are recurrent findings in anterior interosseous nerve syndrome, suggesting neuritis.

In this report, we present and discuss a case of a 43-year-old lady diagnosed with lacertus fibrosus syndrome in the left upper limb and emphasize the stepwise approach to treating the condition while ruling out other differential diagnoses.

## Case presentation

A 43-year-old woman presented to the orthopedics OPD with chief complaints of excruciating pain and tingling sensation in her left upper limb for five weeks, which was insidious in onset, aggravated with elbow extension, and relieved on rest. She also complained of a loss of thumb pinch grip. As per her past history, she is a known case of bronchial asthma, for which she is under medication. The patient was admitted for further evaluation and management. Her physical examination was conducted, which was within the normal range. The patient was cooperative, conscious, and well-oriented to time, person, and place. All systemic examinations were normal.

**Figure 1:**
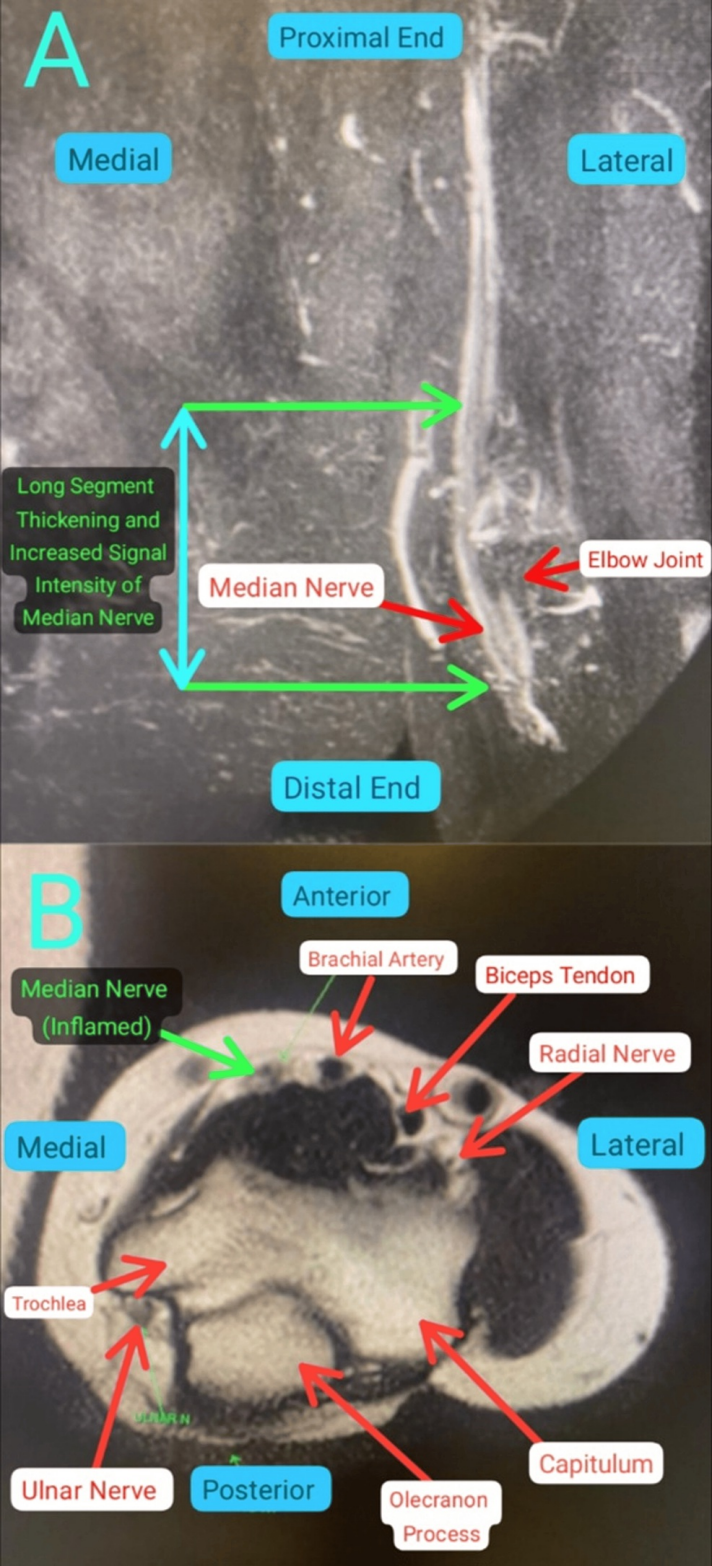
T2-weighted Fat Sat MRI of left elbow joint. (A) Coronal view; (B) axial view show long segment thickening and increased signal intensity of median nerve above and below the elbow joint (Green Arrows) upto the insertion of biceps tendon. About 11 cm of median nerve is inflamed from supracondylar region of humerus to the point where it enters between the two heads of pronator teres. Features are suggestive of median nerve compression neuropathy. Scale bar is 1:1.

After taking due consent, a local examination of the left elbow was done. Wasting of the left thenar muscles was noticed, along with undersensation over the median nerve distribution. There was weakness in the flexor pollicis longus, flexor digitorum profundus, flexor carpi radialis, and thenar muscles of the left hand. The movement of the left elbow was painful and restricted. Bilateral active finger movements were present. Her laboratory reports were normal. Her magnetic resonance imaging (MRI) was done, which showed inflammation of the median nerve proximal to the elbow joint line (Figure 1A-1B). Her nerve conduction study was done as well, which reported normal findings. The team came up with the diagnosis of median nerve compression, but since the exact site of compression could not be found, exploration along the left median nerve course was indicated.

**Figure 2:**
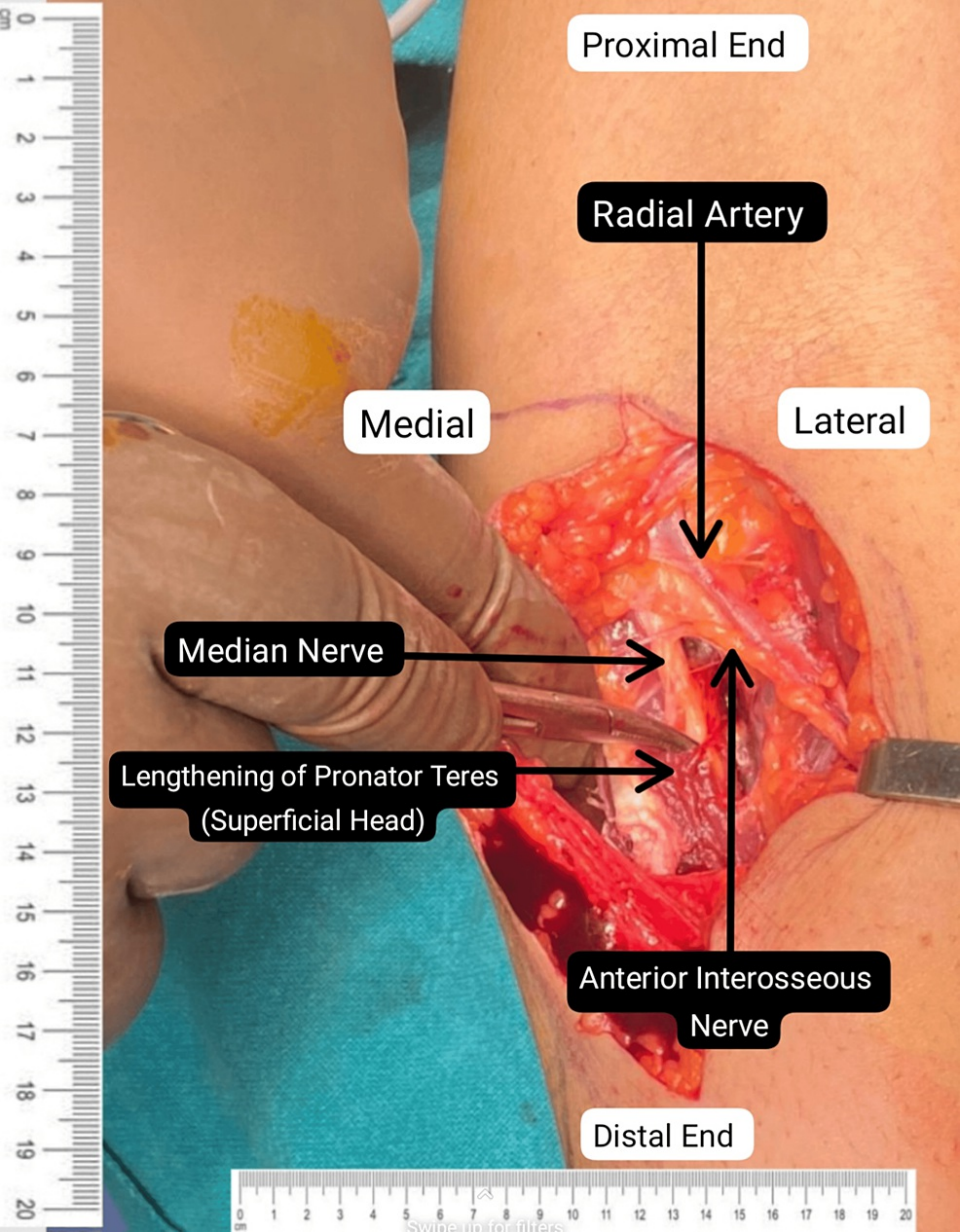
Exploration after lazy 'S' shaped incision over the ventral aspect of the left forearm followed by lengthening of the superficial head of the PT muscle. PT: pronator teres.

**Figure 3:**
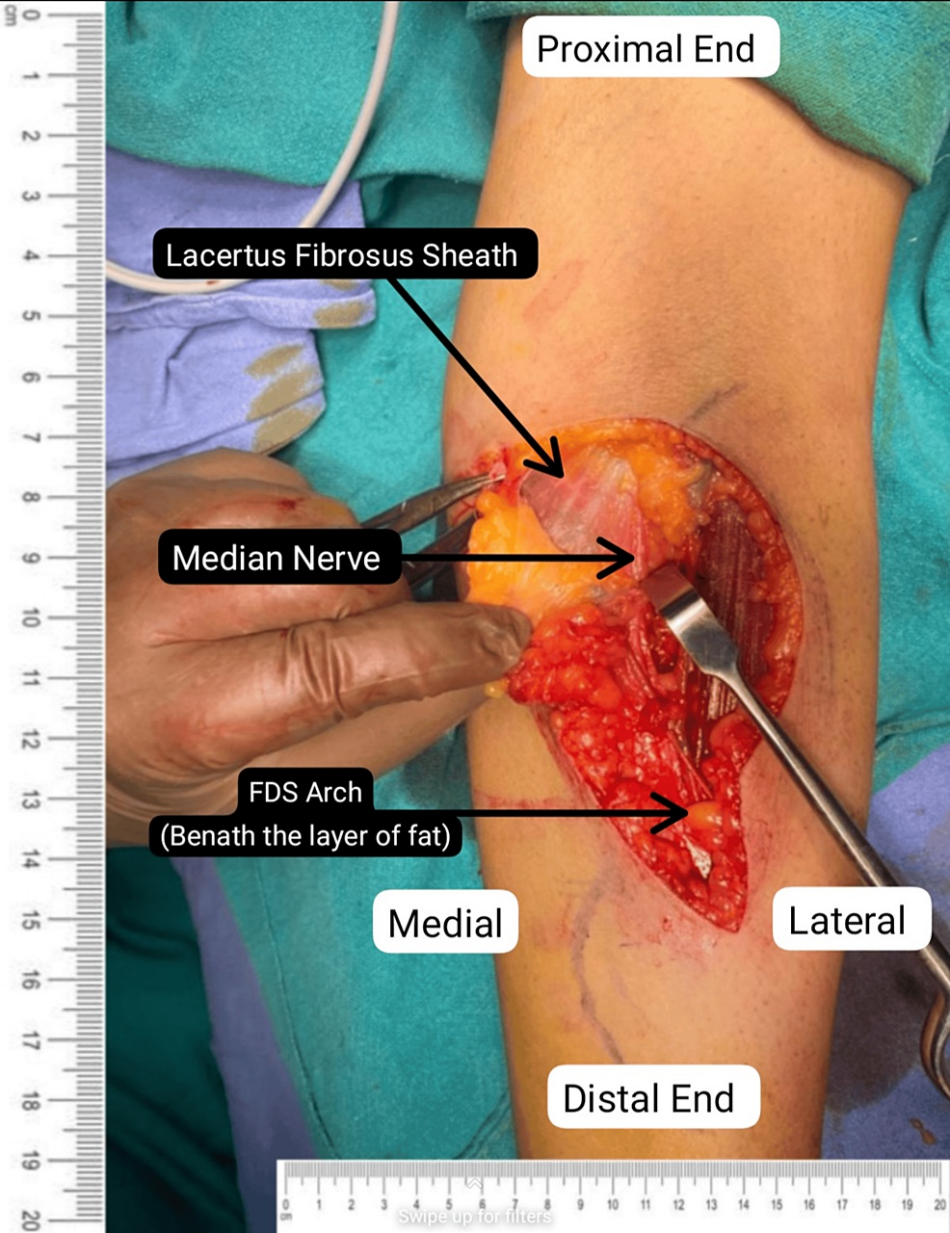
Exposure of lacertus fibrosus sheath and palpation of the FDS arch. FDS: flexor digitorum superficialis.

**Figure 4:**
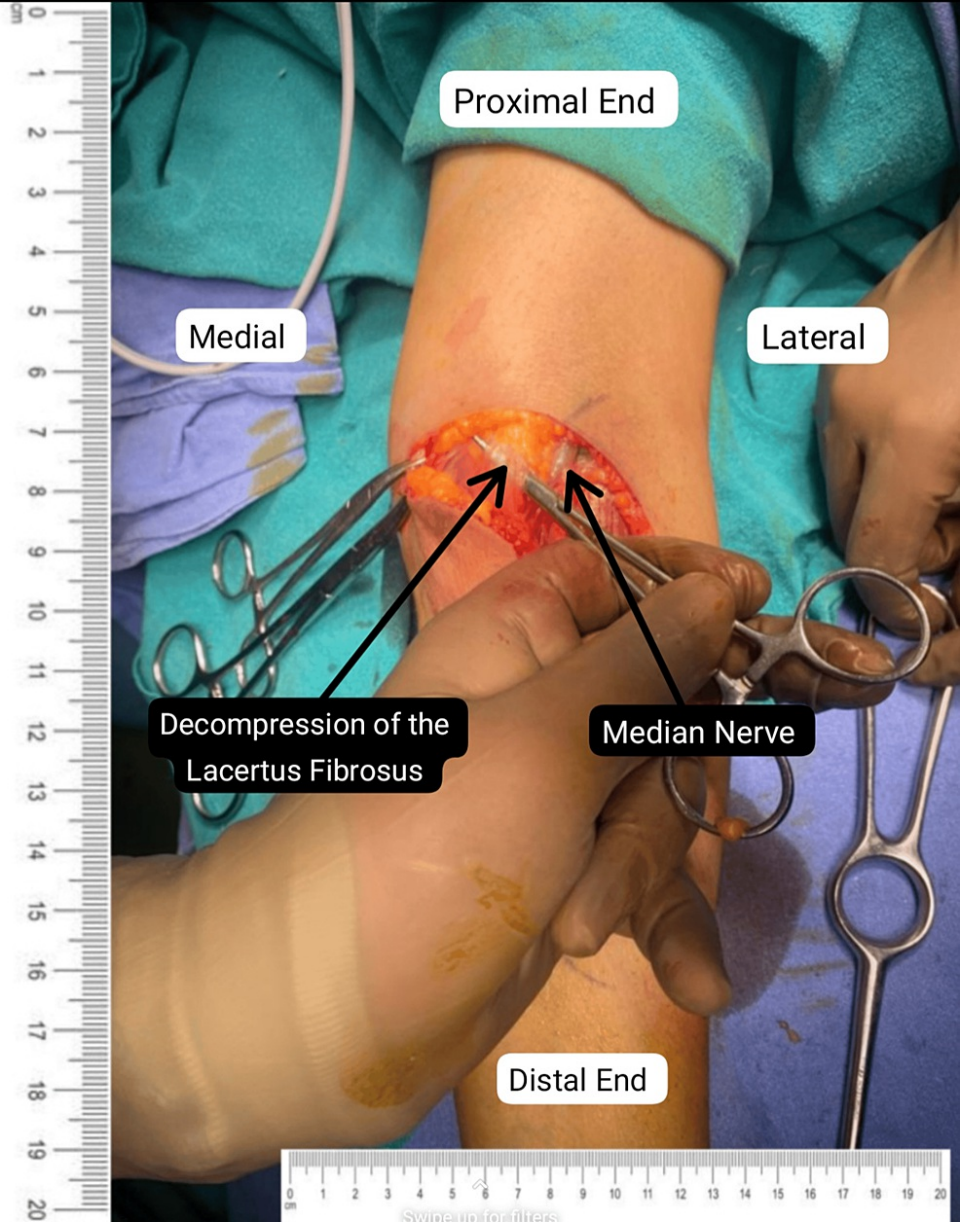
Release of lacertus fibrosus sheath.

**Figure 5:**
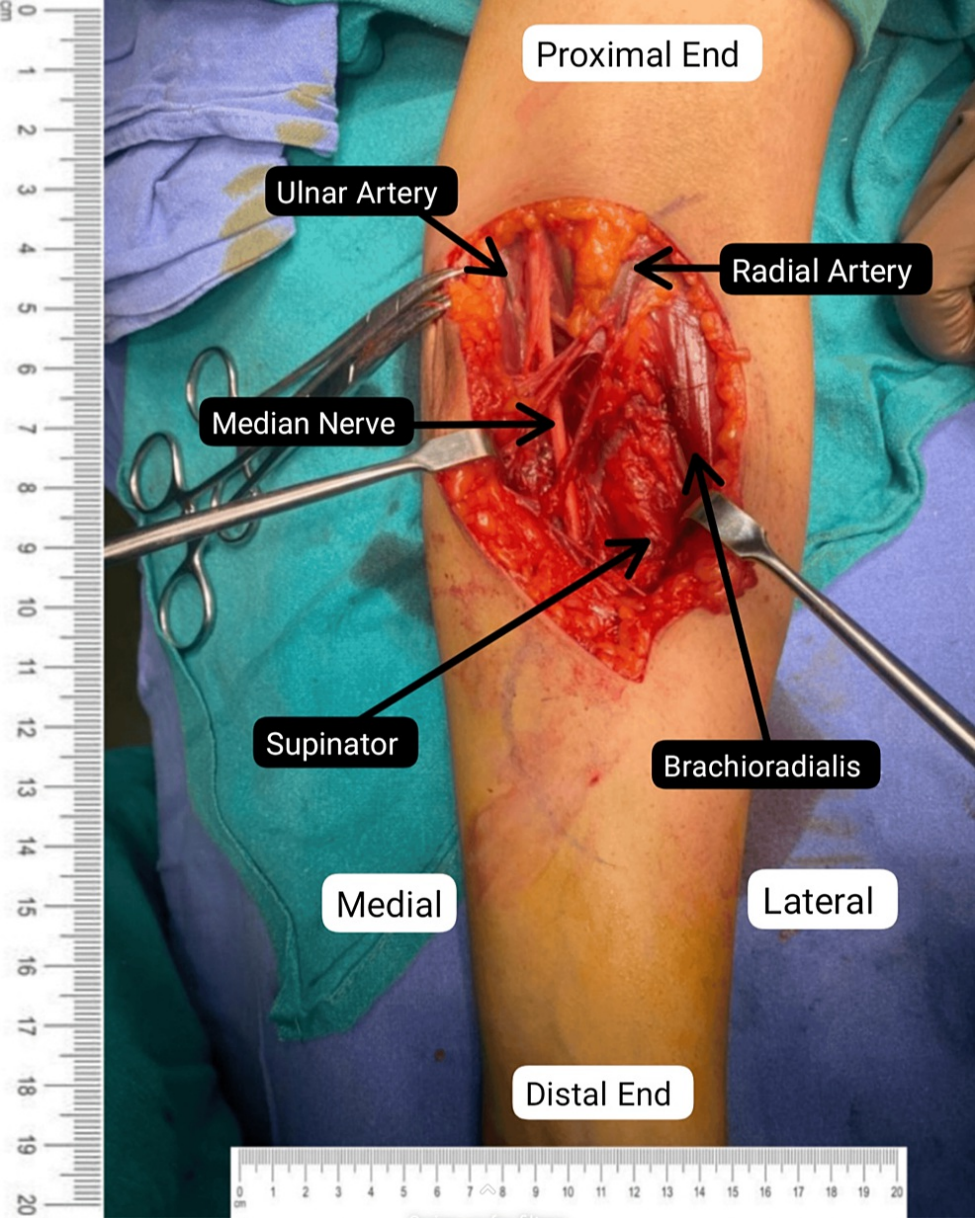
Decompressed median nerve after release of all the potential compression sites.

Informed consent and anesthetic clearance were obtained from the patient for surgery. A lazy "S"-shaped incision was given on the ventral aspect of the left proximal forearm. Exploration was done above the following areas: lacertus fibrosus, pronator teres (PT) muscle, and the flexor digitorum superficialis (FDS) arch. The superficial head of PT was lengthened, and the deep head was divided as per the median nerve (Figure 2). This was followed by palpation of the FDS arch, which was sufficiently spaced (Figure 3). Lastly, a proximal extension of the left elbow joint produced a tight lacertus fibrosus, which was then divided, intending to decompress the left median nerve (Figures 4-5).

The patient tolerated the operation well, and the post-op timeline was uneventful. There was an immediate improvement in the thumb pinch, signifying successful decompression of the left median nerve. The dressing was revised on the first post-op day, and the drain was removed. The patient was ambulatory and discharged in steady condition. The patient was asked to seek medical advice if he encountered a high fever, any discharge, or pain over the surgical site.

## Discussion

**Figure 6:**
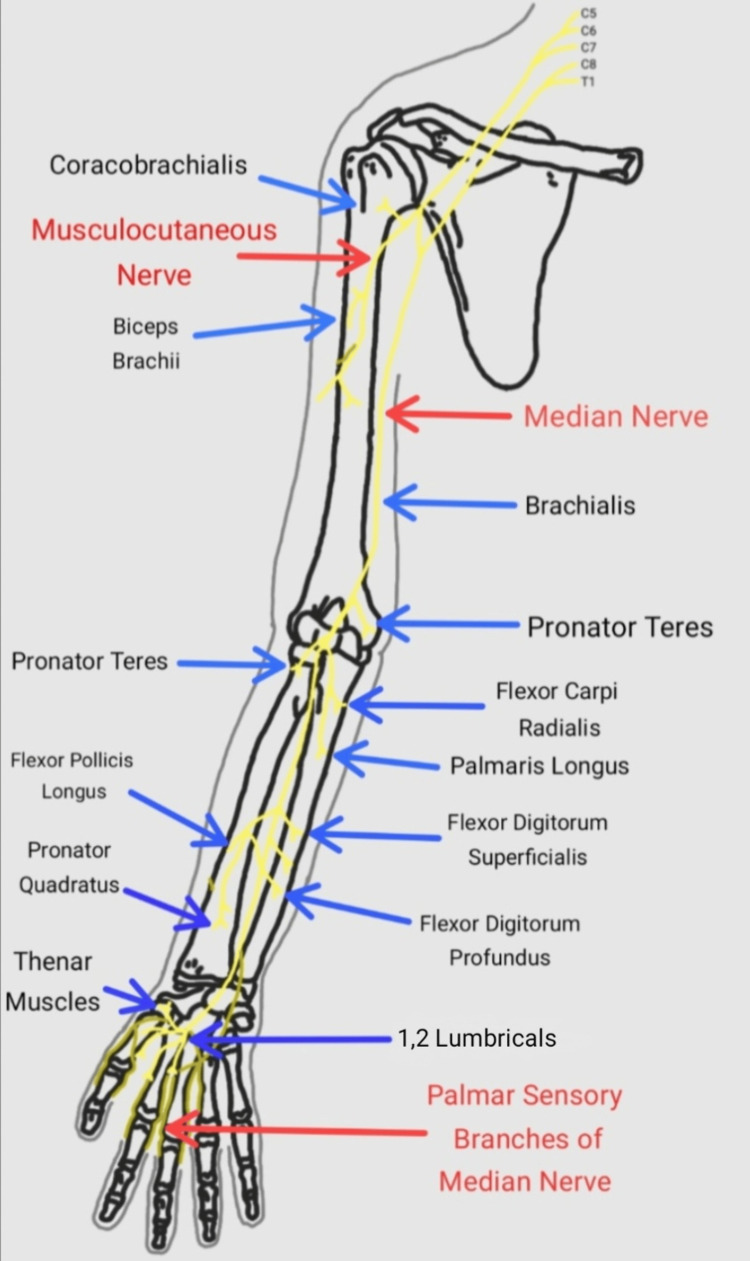
Median nerve and the muscles it supplies in the right upper limb. Route and relations of the median nerve in the right upper limb [14]. Image credit: Pranav Gupta.

The median nerve's typical anatomical relationships in the vicinity of the elbow joint are as follows [14]. In the upper arm, the median nerve descends laterally to the brachial artery. Nearly at the same level as the coracobrachialis, it then moves across the artery from the lateral to the medial side. The nerve then enters the forearm behind the bicipital aponeurosis, commonly known as the lacertus fibrosus, between the two heads of the PT muscle in the cubital fossa (Figure 6). It then moves downward, passing between the FDP and the flexor digitorum superficialis beneath its fibrous bridge. All wrist flexor muscles, excluding the flexor carpi ulnaris and the medial part of the flexor digitorum profundus, are innervated by branches of the median nerve. The anterior interosseous nerve, which emerges in the cubital fossa from the posterior surface of the median nerve, supplies the flexor pollicis longus, the pronator quadratus, and the lateral part of the flexor digitorum profundus.

**Figure 7:**
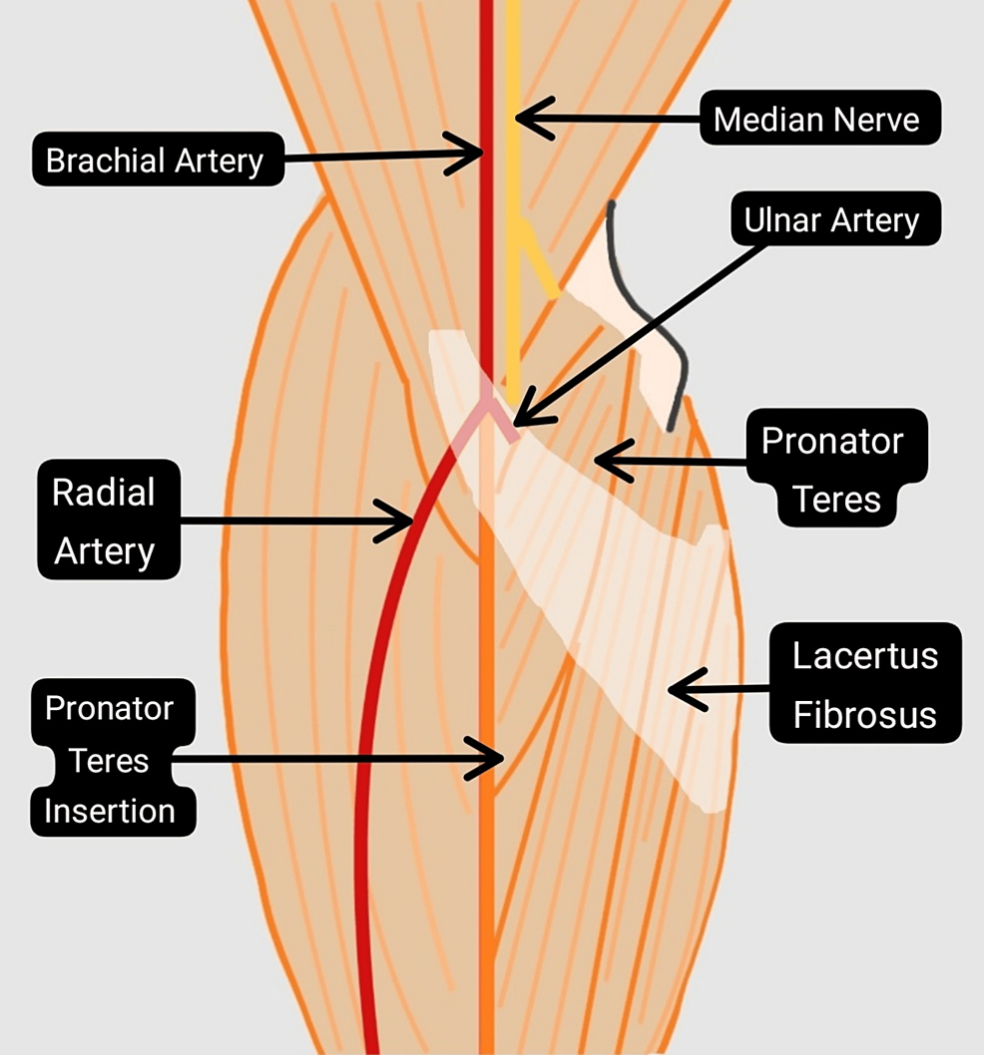
Bicipital aponeurosis in relation to the median nerve. Lacertus fibrosus covers the pronator-flexor mass and the median nerve [16]. Image credit: Pranav Gupta.

The bicipital aponeurosis, also known as the lacertus fibrosus, arises from the biceps brachii and attaches to the pronator-flexor mass fascia (Figure 7). The median nerve and brachial artery pass beneath it, which, during forearm supination, are vulnerable to compression. The median nerve might also be compressed by a persistent median artery [15].

The diagnosis of lacertus fibrosus syndrome is largely predicated on the clinical history and examination of the affected upper limb. It is characterized by a loss of thumb pinch grip, also known as the OK sign. The patient usually complains of pain in the proximal volar forearm, hand, or fingers. Additionally, patients may experience cramping, clumsiness, loss of strength, stiffening of the forearm muscles, aching discomfort, and tonic flexion of the digits [16]. Patients may exhibit positive characteristic findings on USG or MRI, which may also help in locating the site of compression. However, diagnostic investigations may not consistently be as useful in decision-making as clinical assessment.

When operating, all possible areas of pathology, including the PT, lacertus fibrosus, FDS arch, and ligament of Struthers, must be decompressed. If necessary, all fibrotic bands on the same level as the distal arm and proximal forearm ought to be released along the median nerve as well as the anterior interosseous nerve's courses. To obtain a more complete view of the nerve, the PT fascia can be lengthened. After this, the compressive position of the nerve demonstrates diminished vasculature of the vasa nervorum distally and raised tortuosity proximally [17]. In this case, the patient exhibited clinical features pointing towards the diagnosis of median nerve compression on the left upper limb, with uncertainty about the exact site of compression. The majority of cases have compression distal to the elbow. But, in this case, we found the lacertus fibrosus as the offending structure. One clinical point in history and clinical examination that clinched the diagnosis was the aggravation of symptoms with elbow extension. Exploration along the left median nerve was done along with decompression of all the possible compression sites. Instant improvement in thumb pinch grip was noticed, which ensured sufficient decompression of the left median nerve.

The underdiagnosis of lacertus fibrosus syndrome and unsatisfactory results in double-crush syndromes after carpal tunnel release necessitate a thorough examination of this condition's present diagnostic and treatment techniques [18,19]. Treatment guidelines depend on professional views and opinions and ought to be adequately addressed. Randomized studies are required to determine the necessity of surgery in contrast to non-surgical therapy.

## Conclusions

Lacertus fibrosus syndrome is an infrequent median nerve compression disorder generally characterized by impairment of hand strength and hand endurance. Diagnosis is based chiefly on examination and systematic physical examination of the whole upper limb, not only the wrist. A nerve lesion, irrespective of the cause, can heal spontaneously, provided that the nerve stays intact; however, the merits and demerits of surgery must be thoroughly and independently evaluated if no healing ensues. If required, surgical decompression of all the potential compression areas can lead to satisfying results. This report presented available information on the symptoms, diagnosis, treatment, and outcome of lacertus fibrosus syndrome.
